# Macro-morphological characterization and kinetics of *Mortierella alpina* colonies during batch cultivation

**DOI:** 10.1371/journal.pone.0192803

**Published:** 2018-08-07

**Authors:** Xue Fang, Genhai Zhao, Jun Dai, Hui Liu, Peng Wang, Li Wang, Junying Song, Zhiming Zheng

**Affiliations:** 1 Key Laboratory of High Magnetic Field and Ion Beam Physical Biology, Hefei Insitutes of Physical Science, Chinese Academy of Sciences, Hefei, Anhui, China; 2 University of Science and Technology of China, Hefei, China; Institute for Sustainable Plant Protection, C.N.R., ITALY

## Abstract

An effective method for research of macro-morphological characterization and its kinetics was developed by studying the macro-morphological characteristics of *Mortierella alpina*, an oleaginous zygomycete widely used to produce lipids rich in PUFA, in function of culture medium composition and to link morphological features of fungus with the level of lipid production. A number of distinct morphological forms including hollow pellets, fluffy pellets and freely dispersed mycelia were obtained by changing the fermentation factors. By fitting a Logistic curve, the maximum specific growth rate (*μ*_max_)was obtained, which determined the final mycelia morphology. *μ*_max_ of 0.6584 in three kind of morphological forms is the more appropriate. According to the Luedeking-Piret equation fitting, *α*≠0 and *β*≠0, lipid production was partially associated with the hyphal growth, fluffy pellets which turn glucose into lipidwas more effective than the other two kinds of morphological forms.

## Introduction

Microbial lipophilic compounds, called single cell oils (SCO), are potentially interesting to the food and pharmaceutical industries owing to their specific characteristics [1,2]. The most studied target presently carried out in the field is the production of edible oils of special composition or structure, e.g., lipids rich in polyunsaturated fatty acids (PUFA) of medical interest orfat substitutes. Various oleaginous zygomycetes, especially *Mortierella*, have been wildly used as PUFA producing strains [3,4]. Emphasis is mainly put on screening for more effective strains, using suitable substrates, and cultivating the strains under optimum conditions to achieve the maximal yield of PUFA [5–7].

When grown in submerged culture, filamentous fungi are able to develop complex morphologies, which have been classified into three major groups: pellets, mycelia aggregates (so called clumps) and freely dispersed mycelia [8–10]. Studies addressing the morphology and physiology of fungi in liquid cultures have been reviewed [11–13], which showed fungal morphology played a significant role on medium rheology, and thereby affecting the mixing and mass transfer within the bioreactor, but also influenced metabolite activity, resulting in either lower specific growth rate or enhanced enzyme production by strains with altered morphology[10,14,15]. Therefore, the productivity in biotechnological process is often correlated with the morphological form. For example, pellet growth is preferable for pravastatin precursor production by *Pencillium citrinum* [16]and citric acid production by *Aspergillus niger* [17]; dispersed growth is preferable for penicillin production by *Penicillium chrysogenum*[18] and fumaric acid production by *Rhizopus arrhizus* [19]. However, the relationship between fungal morphology and product formation is difficult to investigate, as there are many interrelated factors (viscosity, agitation and DO) encountered in a fermentation system that affect both morphology and desired product production [20–22]. Nevertheless, Bhargava et al. manipulated the fungal growth characteristics to obtain the correct morphology of good performance[23,24].The emergence of molecular biology provides us a powerful tool to fundamentally control mycelia morphology, which represents a simple and inexpensive means of improving production during industrial filamentous-fungal fermentations. Our lab has made some attempts in another filamentous fungus, *Penicillium chrysogenum*, in which the silence of a class III chitin synthase gene led to the formation of small compact pellets and significantly improved penicillin yield [18].

In the case of *Mortierella alpina*, several researchers have reported on the influence of various factors on the morphology and lipid production, such as, dissolved oxygen, mineral addition, the natural nitrogen source etc. [25]. However, there is no thorough report about the morphology formation process and its kinetic analysis under each of morphology during the whole fermentation. In this study, we investigated the macro-morphological characteristics of *Mortierella alpina*in function of culture media composition and made kinetic analysis, trying to reveal the morphogenesis mechanism and link the morphological features with lipid production.

### Nomenclature

Fluffy pellet means that a pellet is mainly composed of radial filaments, just like a chrysanthemum. Radial filaments mean a number of slender and dispersed clumps. Hollow pellet means a hollow sphere that is tightly compacted at the surface with a diameter exceeding 3 mm.

## Material and methods

### Microorganism and media

A strain of *M*. *alpina* I_49_-N_18_, kept in the Key Laboratory of Ion Beam Bioengineering, Chinese Academy of Sciences, was used in this work. The stock culture was maintained on potato dextrose agar (PDA) and was subcultured every 2 months. Spores were washed from fresh PDA plates by deionized water. All treatments were performed in a 250-mL conical flask, containing 50 mL of growth medium (glucose 8%, peptone 1.2%, yeast extract 0.5%) sterilized at 121°C for 20 min and inoculated with 3 mL of spore suspension (10^4^−10^5^/mL). The initial medium pH was adjusted to 8.5 prior to autoclaving at 121°C. The flasks were shaken in an orbital shaker at an agitation rate of 180 rpm and cultured at 28°C for 7 days.

### Fermentation

The carbon source (glucose, sucrose, lactose) and glucose (4%, 6%, 8%, 10%) concentration were studied on the growth medium. The nitrogen source that was added to the culture medium included 20 g/L of peptone, yeast extract, or a mixture of peptone and yeast extract at the different ratio. All samples were incubated for 7 days (stationary phase) to determine the nitrogen source effect. The different C/N ratio was obtained by changing the concentration of mixture of peptone and yeast extract at 2:1 (w/w) from 10 g/L to 30 g/L with a fixed initial glucose concentration at 80 g/L. All cultures were done in triplicate and the results represented as an average value.

### Image analysis

A stereoscopic microscope equipped with a monochrome CCD camera (Olympus Co., Japan) was used for image observation of the mycelia. The captured images were fed into a computer and analyzed using the image analysis software (Patent ID: 2011R11S044664.). The captured images were thresholded to obtain binary images. Opening was then applied to the binary images to improve their qualities. Repeated opening cycles were applied to each pellet until only the pellet core remained. Subtraction of the pellet core from the whole mycelia provided the filamentous part. For each sample, images of at least 20 colonies (pellet + filaments) were used to determine the morphological parameters. The average value was used to analyze the morphological parameters. The projected area of the whole mycelia (A_m_), area of the pellet core (A_pc_),equivalent diameters of the projected area of the whole pellet (D_m_) and the diameter of pellet core were determined by the method developed by Koike *et al*. [26]. The fluffy degree of the pellet was given by A_pc_/A_m_. Fifty elements (defined as either pellets or dispersed filaments) were randomly selected in shake flasks, and then dried at 80°C to get the dry cell weight (DCW). We ideally regarded the mycelia as a regular sphere to obtain its volume. The compactness was determined as follows:

$$compactness=\frac{DCW/50}{\frac{4}{3}\times \pi \times {\left(\frac{D_{m}}{2}\right)}^{3}}$$

### Modeling

In general, the fermentation kinetic model can be subdivided into a growth model, a substrate model and a product model. Among the many unstructured models describing the growth kinetics of microorganisms, the logistic model has been a most popular one due to its “goodness of fit” and has been widely used in describing the growth of microorganism [27, 28]. Based on the logistic model, Luedeking-Piret equation combined growth-associated and non-growth-associated contributions was developed [29].For cell concentration X, the logistic model was derived as follows:

$$\frac{dx}{dt}=\mu_{max}(1-\frac{X}{X_{m}})X$$

μ_max_ is the maximum specific growth rate with respect to the fermentation conditions. Generally, product formation was associated not only with the cell growth but also with the cell concentration, therefore a product formation kinetic equation can be used as follows:

$$\frac{dp}{dt}=\alpha \frac{dx}{dt}+\beta X$$

α, β value represented the degree of correlation lipid accumulation rate with the cell growth rate and cell concentration, respectively. Data fitting was performed using the fitting toolbox of Matlab (Version 2011a).

### Analytical methods

Fungal mycelia were harvested by filtration through four layers of gauze, washed with clean water and dried at 80°C overnight to constant weight to obtain the dry cell weight. Lipids were extracted from the dried fungal cells by the Soxhlet method. Appropriate amount of dried mycelia were ground into a fine powder prior to extraction with petroleum ether for 6–8 h. Total lipid were get indirectly according to the dry cell weight. The residual glucose concentration was determined using SBA-40C glucose analyzer (Shandong Academy of Sciences, China). All data were averages of triplicate determinations.

## Results

### Distinct morphological forms in submerged cultivation

**Fig 1:**
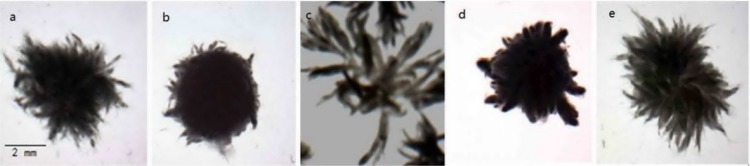
Several distinct macro-morphological forms of *M*. *alpina* under different culture medium. a Fluffy pellets (glucose of 100g/l), b Fluffy pellets cover by slender filamentous (peptone), c Dispersed filaments (yeast extract or corn steep liquor), d Hollow pellets around a little filaments (peptone/yeast extract(3:1)), e Dispersed mycelia (peptone/yeast extract (2:1)). The specific image of mycelia was carried out by the stereoscopic microscope equipped with a monochrome CCD camera at 10X 0.8 times.

During the optimization of fermentation conditions for PUFA production, we found that the culture medium component could influence the macro-morphology of *Mortierella alpina*. Mycelia only grew well on the medium supplemented 80 g/L glucose as the carbon source. Growth defects were observed and morphology was presented in the form of smooth pellets in the application of other carbon source, such as sucrose, lactose, etc. Different initial glucose concentration had a great influence on morphology. Increasing the glucose concentration to 100 g/L, *M*. *alpina* appeared as pellets but the mycelia were slender (Fig 1A).The reason for slender hyphae is that high concentrations of glucose cause excessive osmotic pressure in fermentation broth, which lead to intracellular water molecules leakage, further affecting the mycelial growth. The nitrogen source also influenced the morphology, a variety of morphological forms could be obtained in media containing different nitrogen sources. Mycelia aggregated into densely hollow pellets when peptone was used (Fig 1B), whereas dispersed filaments were observed in the case of yeast extract or corn steep liquor (Fig 1C). Hollow pellets around a little filaments were observed when adjusting the ration of peptone to yeast extract at 3:1 (wt/wt, Fig 1D). Interestingly, anintermediate morphology was obtained adjusting the ration of peptone to yeast extract at 2:1 (wt/wt) in complex nitrogen source, i.e., fluffy pellets growing with radial filaments (Fig 1E).

### Growth and lipid production performance

It was known that the nitrogen source were vital to accumulating microbial lipids. We especially analyzed cell growth and lipid yields of *M*. *alpina* under different morphology caused by different nitrogen sources. The relevant results were listed in Table 1.When peptone was the sole nitrogen source, the biomass was low whereas the lipid content was up to 39.9% in biomass. When the peptone was partially substituted by yeast extract, the morphology progressively changed to filaments from pellets. Meanwhile, the biomass and lipid production exhibited a strong positive correlation. When yeast extract was the sole nitrogen source, the biomass was considerable, but the lipid content was only 25.6% in biomass. Overall, the mixture of peptone and yeast extract (2:1 w/w) induced fluffy pellets and gave the highest biomass and lipid yield. This result indicates that complex nitrogen sources could provide kinds of essential nutrients for cell growth and lipid accumulation, on the other hand, different morphological forms might result in different rheological properties, and therefore had different effects on mixing and mass transfer within the culture, which was an important aspect to affect lipid production as well [30,31].

**Table 1:** Lipid production, biomass and morphology of *M*. *alpina* with different nitrogen sources (means± SD).

Nitrogen sources	DCW (g/L)	Lipid (g/L)	Mycelia morphology
peptone	7.84±1.46	3.13±0.27	hollow pellets
peptone: yeast extract (3:1)	15.18±1.02	6.57±0.42	hollow pellets
peptone: yeast extract (2:1)	25.8±1.85	9.98±0.39	fluffy pellets
peptone: yeast extract (1:1)	28.11±1.59	5.73±0.45	radial filaments
yeast extract	21.06±1.23	5.41±0.32	dispersed filaments

### Characteristic parameters of each morphology

**Fig 2:**
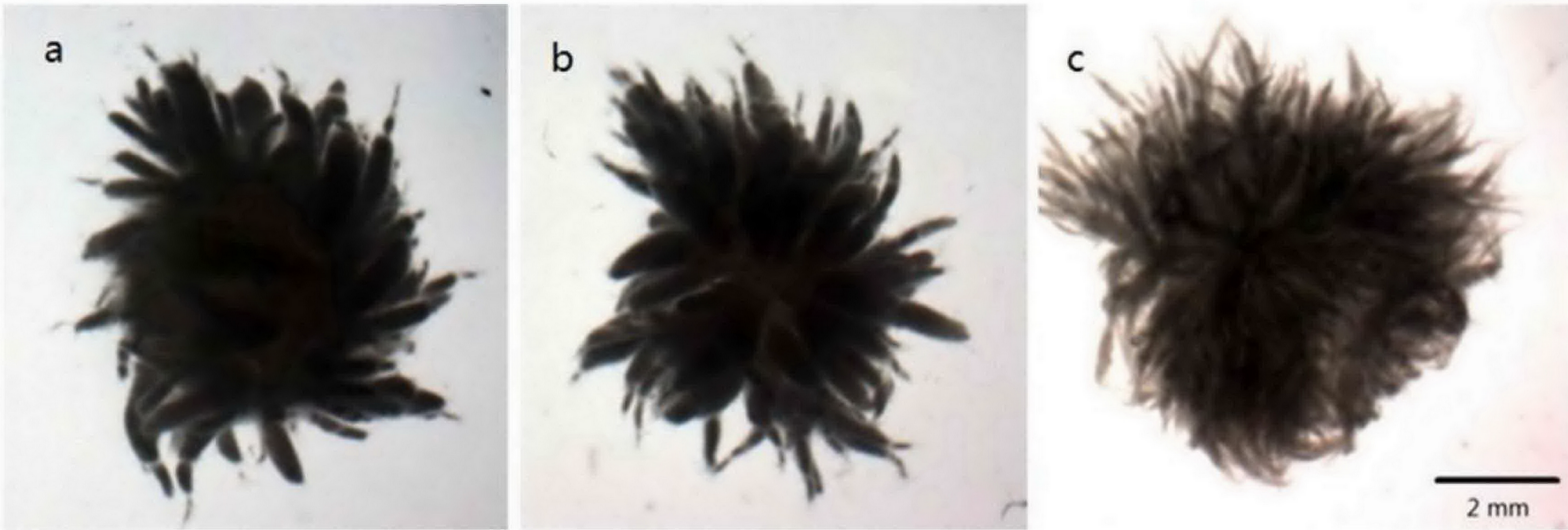
Macro-morphological diversity under the influence of different C/N ratio in a culture medium. a Hollow pellets with some filaments, 10 g/L nitrogen source, b Fluffy pellets, 20 g/L nitrogen source, c Dispersed filaments, 30 g/L nitrogen source. The specific image of mycelia was carried out by the stereoscopic microscope equipped with a monochrome CCD camera at 10X 0.8 times.

As shown above, both carbon and nitrogen sources had great impact on cell growth, mycelia morphology and lipid production. From the production point of view, the C/N ration was a more important factor to influence the intracellular lipid accumulation. The mixture concentration of peptone and yeast extract (2:1 w/w) was varied from 10 to 30 g/L while the glucose concentration was fixed at 80 g/L, correspondingly, the C/N ratio was 28.2, 14.1 and 9.4. Photographs depicting the morphology of mycelia cultured at those C/N rations were shown in Fig 2.With an increase in C/N ratio, macro-morphological changes had a gradual process, i.e., from freely dispersed mycelia to hollow pellets. Each of morphology was characterized and significant features were illustrated in Table 2. The ratio of pellet core area to whole mycelia area were zero in dispersed mycelia, 43.8% in fluffy pellets and 81.1% in hollow pellets. The mean diameter was 6.01 mm in dispersed filaments, 4.41 mm in fluffy pellets and 4.31 mm in hollow pellets. The compactness was significantly higher in fluffy pellets (1.7 fold) and dispersed mycelia (1.53 fold) when compared to hollow pellets. Overall, with the increase of C/N ratio, the ratio of pellet core area to whole mycelia area significantly increased, but the total mycelia diameter, mean projected area and the compactness were just the opposite.

**Table 2:** The detailed morphological characteristic parameters of each morphology (means ± SD).

Nitrogen concentration (g/L)	10	20	30
Morphology	hollow pellets	fluffy pellets	dispersed mycelia
Glucose uptake ratio (%)	55±3.2	97±1.8	90±2.6
A_pc_/ A_m_	0.811	0.438	0
Compactness	42.72±3.16	71.36±5.32	65.77±4.73
Mean projected area (mm^2^)	13.01±0.62	15.27±1.36	21.92±1.84
Diameter (mm)	4.31±0.21	4.41±0.15	6.01±0.34
Diameter of pellet core (mm)	3.88	2.91	0

### Growth process and fermentation characteristics under mycelium morphology

**Fig 3:**
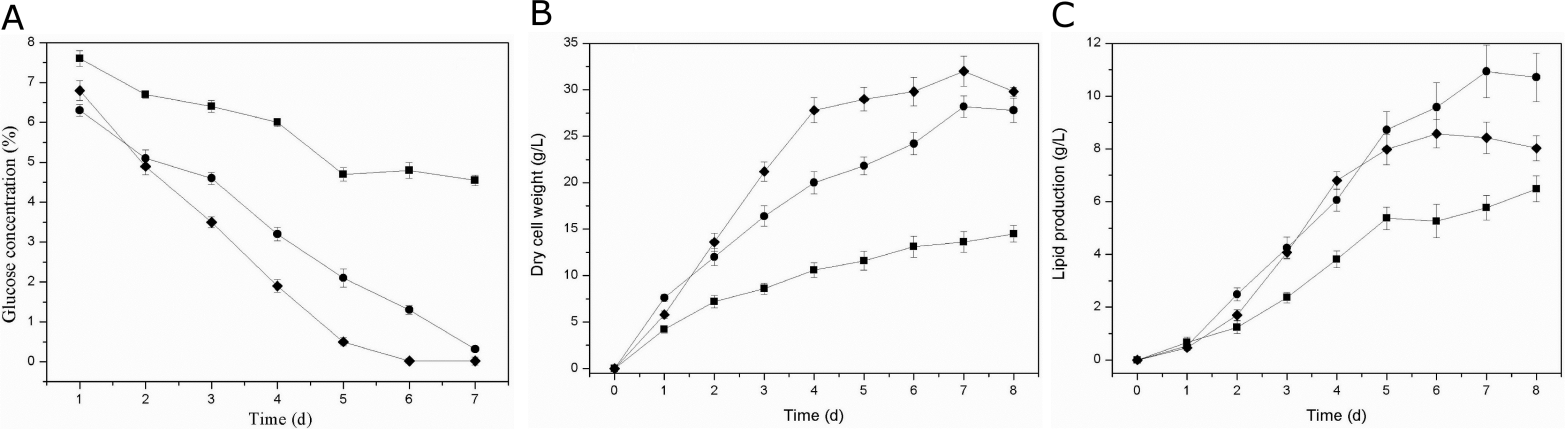
Time course of glucose concentration, dry cell weight (DCW) and lipid production in shake flask cultures inoculated under different morphology. a Glucose concentration, b Dry cell weight, c lipid production. Solid square symbol reflect hollow pellets, solid circle reflect fluffy pellets, solid diamond reflect dispersed filaments.

The time courses for residual glucose concentration, biomass and lipid production in different C/N ratio were shown in Fig 3. Besides the variance in morphology, parameters during the fermentation process were also remarkable different. In the C/N ratio of 28.2, glucose concentration decreased to 45.5 g/Lat the fifth day after inoculation but almost did not consumed in the following days. As for C/N ratio of 14.1, glucose was rapidly consumed at a relatively uniform rate and depleted in the end. The glucose concentration was merely 5 g/L. In the case C/N ratio of 9.4, glucose concentration decreased to 35.0 g/L at the third day and was depleted at the fifth day after inoculation (Fig 3A).The lipid production was determined by biomass and the lipid content in biomass. It achieved a high lipid yields when the biomass and lipid content were both high. As show in Fig 3B, the biomass level was poor for C/N ratio of 28.2 and increased biomass level was obtained in the C/N ratio of 14.1 and 9.4. The final biomass of mycelia with filamentous growth at the ratio of 9.4 was basically the same but its total lipid content (26%) was relatively low when compared to fluffy pellets in the ratio of 14.1(Fig 3B). Therefore, mycelia get the highest lipid production at the C/N ratio of 14.1 with fluffy pelleted growth in Fig 3C.

### Relevant kinetic analysis

It was reported that the higher special growth rate, the less likely the mycelia aggregated together [32]. To confirm the correlation of morphogenesis with the hyphal special growth rate, the fermentation curves in the different C/N ratio were fitted through classical logistical equation and Luedeking-Piret equation. Obtained kinetic parameters were illustrated in Table 3. As shown in Table 3, the specific growth rate μ_max_ was much higher when the C/N ratio was lower. Visible mycelia appeared in the form of small flake-like floc in the first day and turned into freely dispersed filaments at the second day. With a low specific growth rate μ_max_ in the nitrogen concentration of 10 g/L and 20 g/L, mycelia became small spherical floc firstly and aggregated into pellets at the second day. After the specific morphology in all cultures were formed at the second day, the morphology remained unchanged until the end of cultivation. It could be postulated that the mycelia grew quickly with a low branch formation rate and a high tip extension rate at the low C/N ratio.

**Table 3:** The kinetic parameters of fermentation process cultivated with each distinct morphology.

Morphology	*μ*_max_ (d^-1^)	*DCW*_m_ (g· l^-1^)	*Q*_xmax_ (g·l^-1^·d^-1^)	*Q*_Lmax_ (g·l^-1^·d^-1^)	*α*	*β*
Hollow pellets	0.4373	16.19	1.7678	1.0198	0.5594	0.0037
Fluffy pellets	0.6584	28.44	4.6488	1.9557	0.3531	0.0204
Dispersed mycelia	1.153	30.68	8.6831	2.1567	0.2289	0.0127

The detailed cell growth rate (*Q*_X_) and lipid accumulation rate (*Q*_L_) under each of morphology were illustrated in Table 4. For fluffy pellets and dispersed mycelia, *Q*_X_ maintained at a high value in the first four days of the fermentation process. The hollow pellets grew at a rather low *Q*_X_ in the whole process. According to the fitting result by Luedeking-Piret equation for lipid production, α was much larger than β. It indicated that the lipid yields were closely correlated with the cell growth rate, especially in the logarithmic phase. Based on this, the lipid production should be the best in the case of dispersed mycelia. It could also be seen that the *Q*_L_ in dispersed mycelia was larger than the other two morphologies in the first three days indeed. Nevertheless, *Q*_L_ suddenly declined in the next days, which might be the true reason for low lipid production in dispersed mycelia. Comparing α value among different morphological forms, it suggested that the dependence degree of lipid production on the mycelia growth was smaller in dispersed mycelia than that in pellets. Therefore, it was of great importance to control the morphogenesis by adjusting the initial special mycelial growth rate μ at the start stage of cultivation. However, the observed changes in productivity might not be due to the change in morphology alone. The external parameter might have affected the fungal physiology, which could in turn affect the morphology independently.

**Table 4:** The detailed process of the growth rate and lipid accumulation rate at every day for each morphology.

	Hollow pellets		Fluffy pellets		Dispersed mycelia	
Time(d)	*Q*_g_ (g·l^-1^·d^-1^)	*Q*_l_ (g·l^-1^·d^-1^)	*Q*_g_ (g·l^-1^·d^-1^)	*Q*_l_ (g·l^-1^·d^-1^)	*Q*_g_ (g·l^-1^·d^-1^)	*Q*_l_ (g·l^-1^·d^-1^)
1	1.5115	0.864	3.42	1.3471	5.5319	1.3421
2	1.7115	0.9818	4.4093	1.7771	8.6831	2.1567
3	1.7678	1.0198	4.6488	1.9557	7.3244	1.953
4	1.661	0.9663	3.9668	1.8041	3.6159	1.1734
5	1.4275	0.8414	2.822	1.4694	1.346	0.6835
6	1.1361	0.6831	1.761	1.141	0.4489	0.4886
7	0.8503	0.5269	1.0116	0.9041	0.1442	0.4223
8	0.6077	0.3939	0.5539	0.758	0.0458	0.4008

## Discussion

It was interesting to find several morphological forms during the optimization of fermentation conditions. The kind and concentration of nitrogen source in the medium had a key role in the formation of mycelia morphology. Three types of morphology were obtained, i.e. hollow pellets, fluffy pellets and dispersed filaments through adjusting the mixture ratio and total concentration of yeast extract and peptone. When the peptone was partially substituted by yeast extract, the morphology gradually changed to filaments from pellets. We got the same three morphologies in the experiment of different C/N ratio. It can be concluded that the morphogenesis must be related to the amount of yeast extract. Park et al [33]also reported that the different nitrogen source could induce circular pellet and radial filamentous mycelia during the culture of *Mortierella alpina*.

In our study, we described in detail the fermentation characteristics in the different C/N ratio at a fixed mixture ratio of peptone and yeast extract and quantitatively analyzed its characteristics. The prototype of morphology appeared at the first day after inoculation in both culture and the macro-morphology did not change until the end of fermentation. At present, it is not clear how the morphology was formed. However, the morphogenesis must be related to the germination of spore and early growth of hypha. Park et al [33]reported that macro-morphology could be predicted based on the calculated micro-morphological parameters of early growth mycelium using the flow-through chamber. Thomas reported that the morphology can change with the specific growth rate [34]. The specific growth rate of the fungus also can be changed by the use of different substrates, because different uptake systems are involved, the metabolism of the cell will change, and this may indirectly affect the morphology [35].

Then the fermentation characteristics of *M*. *alpina* with those adjusted morphology were kinetically analyzed using classical logistical equation and Luedeking-Piret equation. Results showed that the kinetic parameters differed from each other. We assessed the specific kinetic parameters of fermentation process, linked them with the morphogenesis and lipid accumulation after inoculation. To some extent, the specific growth rate μ_max_ reflected the hyphal growth rate after germination of spore. The higher the μ_max_ is, early mycelia may grow at a high branch rate and tip extension rate. According to the fitting result of Luedeking-Piret equation, the lipid accumulation rate is closely related to the mycelia growth rate. It is different from the reported that the lipid only accumulated when the celluar growth is inhabited due to the nitrogen exhaustion [36,37]. The reason was perhaps the different strains. The different induced macro-morphology at the second day must play an important role in the lipid accumulation. As the mycelia aggregated so tightly, it is difficult to understand which micro-morphology of mycelia was suitable for the lipid accumulation. Whereas, it is easy to understand how mycelia macro-microbiology effect the rheology of culture broth, the mass transfer and oxygen transfer. The hollow pellets was formed because its oversized pellet core diameters (3.88 mm) exceeded the maximum distance of efficient nutrient diffusion. Therefore, the mycelial growth was restricted due to substrate limitation in the interior of the dense pellet core, resulting in low DCW. Molecular diffusion can take place easily throughout the less dense pellets, i.e., fluffy pellets and dispersed filaments via turbulent diffusion and convective flow. But the filamentous growth can lead to highly viscous broths with non-Newtonian, pseu-doplastic flow behavior. Overall, fluffy pellets with radial filaments had an appropriate μ_max_ and good behavior on broth could be introduced as the optimal morphology of *M*. *alpina* for producing the microbial oils.

## Conclusions

The maximum specific growth rate (*μ*_max_) is closely related to the formation of morphology, lipid production was partially associated with the hyphal growth. Mycelia with different morphologies utilized glucose based different metabolic pathways. Dispersed mycelia produced ingredients other than lipid by secondary metabolism, whereas fluffy pellets turn glucose more efficiently into lipid. The present study provides some valuable information about morphological control related to nutritional ingredient and kinetic analyses of whole fermentation process in submerged cultivation.

## Supporting information

S1 Fig Several distinct macro-morphological forms of *M. alpina* under different culture medium. The specific image of mycelia was carried out by the stereoscopic microscope equipped with a monochrome CCD camera at 10X 0.8 times.
(DOCX)

S2 Fig Macro-morphological diversity under the influence of different C/N ratio in a culture medium. The specific image of mycelia was carried out by the stereoscopic microscope equipped with a monochrome CCD camera at 10X 0.8 times.
(DOCX)

S3 Fig Time course of glucose concentration, dry cell weight (DCW) and lipid production in shake flask cultures inoculated under different morphology. Solid square symbol reflect hollow pellets, solid circle reflect fluffy pellets, solid diamond reflect dispersed filaments. Results are representative of at least three independent experiments.
(DOCX)

S1 Table Lipid production, biomass and morphology of *M. alpina* with different nitrogen sources. Results are representative of at least three independent experiments(means± SD).
(DOCX)

S2 Table The detailed morphological characteristic parameters of each morphology. Results are representative of at least three independent experiments (means± SD).
(DOCX)

S3 Table The kinetic parameters of fermentation process cultivated with each distinct morphology. Based on the logistic model Luedeking-Piret equation, Data fitting was performed using the fitting toolbox of Matlab (Version 2011a).
(DOCX)

S4 Table The detailed process of the growth rate and lipid accumulation rate at every day for each morphology. Based on the logistic model Luedeking-Piret equation, Data fitting was performed using the fitting toolbox of Matlab (Version 2011a).
(DOCX)
