# Supplementary material for: Macro-morphological characterization and kinetics of Mortierella alpina colonies during batch cultivation
Source: PLoS One. 2018 Aug 7;13(8):e0192803. doi: 10.1371/journal.pone.0192803 (PMC6080745; doi:10.1371/journal.pone.0192803)
Supplement: S1 Fig — The specific image of mycelia was carried out by the stereoscopic microscope equipped with a monochrome CCD camera at 10X 0.8 times. (DOCX) [file pone.0192803.s001.docx]

S1 Fig. Several distinct macro-morphological forms of *M. alpina* under different culture medium. The specific image of mycelia was carried out by the stereoscopic microscope equipped with a monochrome CCD camera at 10X 0.8 times.

(TIF)
