# Supplementary material for: Macro-morphological characterization and kinetics of Mortierella alpina colonies during batch cultivation
Source: PLoS One. 2018 Aug 7;13(8):e0192803. doi: 10.1371/journal.pone.0192803 (PMC6080745; doi:10.1371/journal.pone.0192803)
Supplement: S3 Fig — Solid square symbol reflect hollow pellets, solid circle reflect fluffy pellets, solid diamond reflect dispersed filaments. Results are representative of at least three independent experiments. (DOCX) [file pone.0192803.s003.docx]

S3 Fig. Time course of glucose concentration, dry cell weight (DCW) and lipid production in shake flask cultures inoculated under different morphology. Solid square symbol reflect hollow pellets, solid circle reflect fluffy pellets, solid diamond reflect dispersed filaments. Results are representative of at least three independent experiments.

(TIF)
