# Supplementary material for: Macro-morphological characterization and kinetics of Mortierella alpina colonies during batch cultivation
Source: PLoS One. 2018 Aug 7;13(8):e0192803. doi: 10.1371/journal.pone.0192803 (PMC6080745; doi:10.1371/journal.pone.0192803)
Supplement: S1 Table — Results are representative of at least three independent experiments(means± SD). (DOCX) [file pone.0192803.s004.docx]

S1 Table. Lipid production, biomass and morphology of *M. alpina* with different nitrogen sources. Results are representative of at least three independent experiments(means± SD).
