# Supplementary material for: Macro-morphological characterization and kinetics of Mortierella alpina colonies during batch cultivation
Source: PLoS One. 2018 Aug 7;13(8):e0192803. doi: 10.1371/journal.pone.0192803 (PMC6080745; doi:10.1371/journal.pone.0192803)
Supplement: S2 Table — Results are representative of at least three independent experiments (means± SD). (DOCX) [file pone.0192803.s005.docx]

S2 Table. The detailed morphological characteristic parameters of each morphology. Results are representative of at least three independent experiments (means± SD).
