# Supplementary material for: Macro-morphological characterization and kinetics of Mortierella alpina colonies during batch cultivation
Source: PLoS One. 2018 Aug 7;13(8):e0192803. doi: 10.1371/journal.pone.0192803 (PMC6080745; doi:10.1371/journal.pone.0192803)
Supplement: S4 Table — Based on the logistic model Luedeking-Piret equation, Data fitting was performed using the fitting toolbox of Matlab (Version 2011a). (DOCX) [file pone.0192803.s007.docx]

S4 Table. The detailed process of the growth rate and lipid accumulation rate at every day for each morphology. Based on the logistic model Luedeking-Piret equation, Data fitting was performed using the fitting toolbox of Matlab (Version 2011a).
